# Factors affecting the use of prenatal care by non-western women in industrialized western countries: a systematic review

**DOI:** 10.1186/1471-2393-13-81

**Published:** 2013-03-27

**Authors:** Agatha W Boerleider, Therese A Wiegers, Judith Manniën, Anneke L Francke, Walter LJM Devillé

**Affiliations:** 1 Netherlands Institute for Health Services Research (NIVEL), P.O. Box 1568, 3500 BN, , Utrecht, The Netherlands; 2 Department of Midwifery Science, AVAG and the EMGO Institute for Health and Care Research, VU University Medical Center, Amsterdam, The Netherlands; 3 Department of Public and Occupational Health, EMGO Institute for Health and Care Research, VU University Medical Center, Van der Boechorststraat 7, 1081BT, Amsterdam, Netherlands; 4 Faculty of Social and Behavioural Sciences, University of Amsterdam, Oudezijds Achterburgwal 185, 1012DK, Amsterdam, The Netherlands; 5 National Knowledge and Advisory Center on Migrants, Refugees and Health (Pharos), Herenstraat 35, 3507LH, Utrecht, The Netherlands

## Abstract

**Background:**

Despite the potential of prenatal care for addressing many pregnancy complications and concurrent health problems, non-western women in industrialized western countries more often make inadequate use of prenatal care than women from the majority population do. This study aimed to give a systematic review of factors affecting non-western women’s use of prenatal care (both medical care and prenatal classes) in industrialized western countries.

**Methods:**

Eleven databases (PubMed, Embase, PsycINFO, Cochrane, Sociological Abstracts, Web of Science, Women’s Studies International, MIDIRS, CINAHL, Scopus and the NIVEL catalogue) were searched for relevant peer-reviewed articles from between 1995 and July 2012. Qualitative as well as quantitative studies were included. Quality was assessed using the Mixed Methods Appraisal Tool. Factors identified were classified as impeding or facilitating, and categorized according to a conceptual framework, an elaborated version of Andersen’s healthcare utilization model.

**Results:**

Sixteen articles provided relevant factors that were all categorized. A number of factors (migration, culture, position in host country, social network, expertise of the care provider and personal treatment and communication) were found to include both facilitating and impeding factors for non-western women’s utilization of prenatal care. The category demographic, genetic and pregnancy characteristics and the category accessibility of care only included impeding factors.

Lack of knowledge of the western healthcare system and poor language proficiency were the most frequently reported impeding factors. Provision of information and care in women’s native languages was the most frequently reported facilitating factor.

**Conclusion:**

The factors found in this review provide specific indications for identifying non-western women who are at risk of not using prenatal care adequately and for developing interventions and appropriate policy aimed at improving their prenatal care utilization.

## Background

Prenatal care has the potential to address many pregnancy complications, concurrent illnesses and health problems [1]. An essential aspect of prenatal care models concerns the content of prenatal care, which is characterized by three main components: a) early and continuing risk assessment, b) health promotion (and facilitating informed choice) and c) medical and psychosocial interventions and follow-up [2,3]. Another essential aspect of prenatal care models concerns the number and timings of prenatal visits. While there is overall agreement on the importance of early initiation of prenatal care, the number of prenatal visits has led to a great deal of discussion. A Cochrane review of ten RCTs among mostly low-risk women concluded that the number of prenatal visits could be reduced without increasing adverse maternal and perinatal outcomes, although women in developed countries might be less satisfied with this reduced number of prenatal visits [4].

Despite universal healthcare insurance coverage in most industrialized western countries, studies in these countries have shown that non-western women make inadequate use of prenatal care. They are less likely to initiate prenatal care in good time [3,5-7], attend all prenatal care appointments [8] and attend prenatal classes [9]. Furthermore, non-western women have also been shown to be at increased risk for adverse perinatal outcomes. A meta-analysis by Gagnon *et al.* showed that Asian, North African and sub-Saharan African migrants were at greater risk of feto-infant mortality than ‘majority’ populations in western industrialized countries, with adjusted odds ratios of 1.29, 1.25 and 2.43 respectively. This study also found that Asian and sub-Saharan African migrants are at greater risk of preterm birth, with adjusted odds ratios of 1.14 and 1.29 respectively [10]. Besides an increased risk for adverse perinatal outcomes, non-western women are also at increased risk of adverse maternal outcomes, in terms of both mortality [11,12] and morbidity [13].

A few studies have implied a relationship between non-western women’s higher risk of adverse pregnancy outcomes and their use of prenatal care. In a Dutch study conducted by Alderliesten *et al.*, late start of prenatal care was one of the maternal substandard care factors of perinatal mortality that were more common among Surinamese and Moroccan women [14]. In a French study conducted by Philibert *et al.*, the excess risk for postpartum maternal mortality among non-western women was associated with a poorer quality of care, suggesting attention should be paid to early enrolment in prenatal care [15]. This relationship emphasizes the importance of proper use of prenatal care to address pregnancy complications, concurrent illnesses and health problems.

Two previously conducted reviews provide relevant insights into the factors affecting prenatal care utilization [16,17]. The first review focused on women, irrespective of origin, in high-income countries. Ethnicity, demographic factors, socioeconomic factors at the individual and neighbourhood level, health behaviour and provider characteristics were found to be determinants of inadequate prenatal care utilization [16]. The second review focused on first-generation migrant women of western and non-western origin in western industrialized countries. In this review, being younger than 20, poor or fair language proficiency and socioeconomic factors were reported to affect prenatal care utilization [17].

A review specifically focused on factors affecting prenatal care utilization by non-western women, irrespective of generation, was still lacking. Furthermore, qualitative studies -, which are well suited to exploring the experiences and perceptions that play a role in women’s prenatal care utilization - were not included in previously conducted reviews. Also, these reviews were not restricted to countries with similar accessibility to healthcare, which complicates generalization of the results found. In this review, we therefore aimed to identify and summarize all reported factors, irrespective of study design, affecting non-western women’s use of prenatal care and prenatal classes in industrialized western countries with universal insurance coverage. Prenatal (or antenatal) care was defined as all care given by professionals to monitor women’s pregnancy. All courses preparing pregnant women for birth or teaching them how to feed and take care of their baby were defined as prenatal or antenatal classes. ‘Factors’ were defined as all experiences, needs, expectations, circumstances, characteristics and health beliefs of non-western women.

## Methods

### Search strategy

The following databases were searched: PubMed, Embase, PsycINFO, Cochrane, Sociological Abstracts, Web of Science, Women’s Studies International, MIDIRS, CINAHL, Scopus and the NIVEL catalogue. The search was limited to articles published between 1995 and July 2012.

The search strategy consisted of a number of Medical Subject Headings (MeSH) terms and text words, aiming to include as many relevant papers as possible (Additional file 1). It was devised for use in PubMED, and was adapted for use in the other databases. The search was performed in all fields of PubMed (the main database) and in titles, abstracts and keywords for the other databases. No language restriction was applied.

### Methods of screening and selection criteria

The initial screening of articles was based on titles, and the second based on titles and abstracts. Finally, the full texts of the articles were assessed for inclusion. Screening was done by five reviewers (WD, AF, TW, JM, AB). Each article was screened by two reviewers: one of the first four reviewers plus the fifth reviewer. For each article, any discrepancy between the two reviewers was resolved through discussion.

The aim was to identify studies analysing or exploring factors affecting the use of prenatal care by non-western women in industrialized western countries. We therefore included studies if they (a) concerned prenatal care; (b) concerned factors affecting the use of prenatal care; (c) did not concern specific diseases during prenatal care, with the exception of pregnancy-related or postpartum conditions; (d) concerned industrialized western countries (high-income OECD countries except for Japan and Korea) with universal insurance coverage (resulting in exclusion of the USA); (e) concerned non-western women as clients (women from Turkey, Africa, Latin-America, Asia), with results presented at subgroup level; (f) did not concern illegal immigrants, refugees, asylum seekers, students or migrant farm workers (seasonal workers, internal migration); (g) were based on primary research (qualitative, quantitative, mixed methods or case studies).

We have used the term ‘non-western’ women to mean immigrant women from the countries mentioned above, as well as their (immediate) descendants. Studies focusing on women from non-migrant ethnic minority groups (e.g. Aboriginals) were excluded.

In the first two screening stages (titles and titles plus abstracts), studies were included when both reviewers agreed they were eligible for inclusion, or if there was doubt about whether or not to exclude them. In the final screening stage (full texts), studies were included when both reviewers felt they met all the inclusion criteria.

### Data extraction and quality appraisal

The following information was abstracted from the included studies:

(a) general information: authors, journal, publication date, country, language; (b) research design: qualitative, quantitative or mixed-methods design; (c) research population: ethnic group, immigrant generation, sampling method, sample size; (d) analytical approach; (e) all possible factors affecting the use of prenatal care; (f) results and conclusions.

The quality of the studies was assessed by two reviewers, using the Mixed Methods Appraisal Tool (MMAT-version 2011) [18]. This quality appraisal tool seems appropriate as it was designed to appraise complex literature reviews consisting of qualitative, quantitative and mixed-methods studies. Quantitative and qualitative studies are each appraised by four criteria with overall scores varying from 0% (no criterion met) to 100% (all four criteria met). For criteria partially met, we decided to give half of the criterion score. For mixed methods studies, three components are appraised: the qualitative component, the quantitative component and the mixed methods component. The overall score is determined by the lowest component score.

### Synthesis

**Figure 1:**
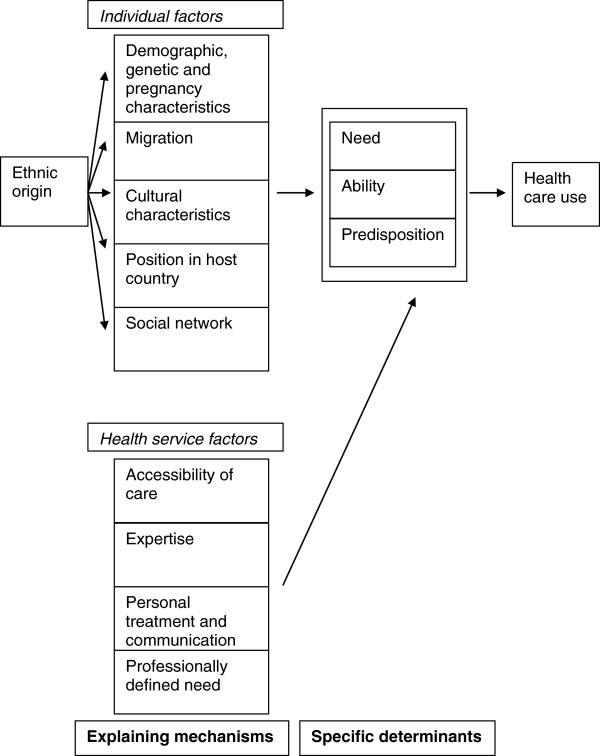
The conceptual framework of Foets et al.

Because of the heterogeneity in terms of countries, non-western groups and methods of analysis, we chose not to conduct a meta-analysis for the quantitative results. Instead, we chose to produce a narrative synthesis of the results of the studies included. For that synthesis, we used the conceptual framework of Foets *et al.* 2007, an elaborated version of Andersen’s healthcare utilization model (Figure 1) [19]. As this conceptual framework integrates the possible explanations for the relationship between ethnicity and healthcare use, it seemed the most appropriate. In this elaborated model the predisposing, enabling and need factors of Andersen are explained by two groups of underlying factors: individual factors and health service factors. The individual factors are subdivided into several categories: demographics and genetics, migration, culture, the position in the host country and social network. The health service factors are subdivided into: accessibility, expertise, personal treatment and communication, and professionally defined need. To fit the factors emerging from the data extraction, the category “demographics and genetics” was expanded to include pregnancy. This finally resulted in the following categories:

#### Individual factors

1) Demographics, genetics and pregnancy: women’s age, parity, planning and acceptance of pregnancy, pregnancy related health behaviour and perceived health during pregnancy

2) Migration: women’s knowledge of/familiarity with the prenatal care services/system, experiences and expectations with prenatal care use in their country of origin, pregnancy status on arrival in the new industrialized western country

3) Culture: women’s cultural practices, values and norms, acculturation, religious beliefs and views, language proficiency, beliefs about pregnancy and prenatal care

4) Position in the host country: women’s education level, women’s pregnancy-related knowledge, household arrangement, financial resources and income

5) Social network: size and degree of contact with social network, information and support from social network

#### Health service factors

6) Accessibility: transport, opening hours, booking appointments, direct and indirect discrimination by the prenatal care providers

7) Expertise: prenatal care tailored to patients’ needs and preferences

8) Treatment and communication: communication from prenatal care providers to women, personal treatment of women by prenatal care providers, availability of health promotion/information material, use of alternative means of communication

9) Professionally defined need: referral by general practitioners and other healthcare providers to prenatal care providers

## Results

**Figure 2:**
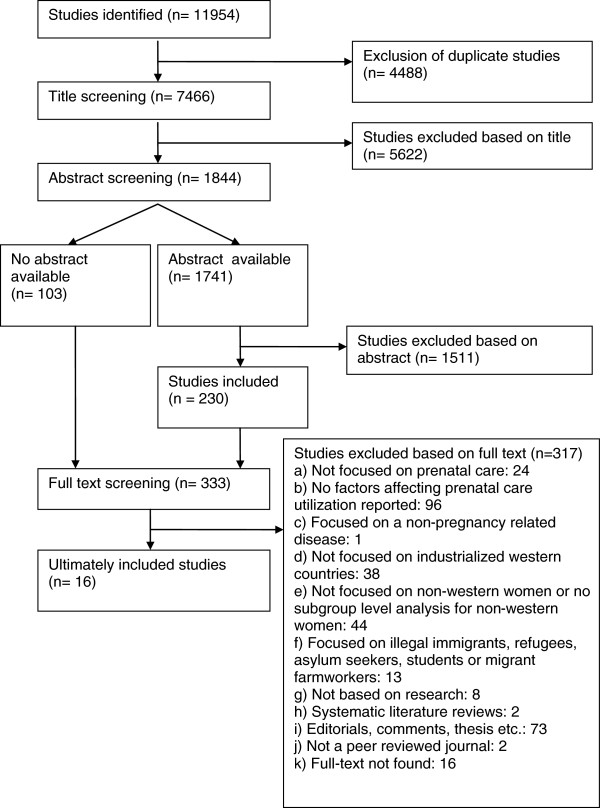
Schematic draft of the selection process.

A total of 11954 articles were initially identified, of which 4488 were duplicates. Title screening of the remaining 7466 non-duplicate references resulted in 1844 relevant articles being selected for abstract screening. After abstract screening, 333 articles were selected for full text screening, either because they were relevant (230) or no abstract was available (103). Finally, full text assessment resulted in 16 peer-reviewed articles being included and their methodological quality being assessed (Figure 2).

### Characteristics of the included studies

Additional file 2 provides an overview of the articles included. Three articles described quantitative observational studies: 2 cohort studies [20,21] and 1 cross-sectional study [22] with methodological quality scores varying between 75% and 100%.

Twelve articles described qualitative studies: seven individual interview studies [23-29], two focus group studies [30,31], two studies combining individual interviews and focus group interviews [32,33] and one study combining individual interviews and observations [34]. The methodological quality scores of eleven of these twelve qualitative studies varied between 50% and 100%, with the twelfth study scoring 25%.

One study used mixed methods - combining a retrospective cohort design with focus groups [35]. Only the focus group yielded relevant information for this review. The methodological quality score of this study was 25%.

The studies were conducted in various industrialized western countries. Nine studies were conducted in a European country [20,21,23,28,29,31-33,35], four in Canada [22,25,27,30] and three in Australia [24,26,34].

Fourteen articles were published in English [20-22,24-34], one in German [23] and one in Italian [35].

The studies included women from different regions of the world. Three studies reported factors for *sub-Saharan African women*: Somali or Ghanaian [29,32,33]; eight for *Asian women*: South Asian [22], Sri Lankan [23], Filipino [26], Vietnamese [27], Indian [30], Thai [34] or a mixture of Asian origins [24,28]; and two for *Turkish women*[21,31]. One study reported factors for Muslim women not further specified [25].

Some studies reported factors for various non-western ethnic groups. One study reported factors for *sub Saharan African women* (Ghanaian), *North African women* (Moroccan), *Turkish women* and other non-western women not further specified [20]. Another study reported factors for *North African women* (Northwest African women) and *Asian women* (Chinese) as part of a group of migrant women [35].

### Barriers to prenatal care utilization

All factors impeding the use of prenatal care were classified as barriers. The first column of Table 1 gives an overview of these factors according to the conceptual framework of Foets *et al.*. Both quantitative and qualitative studies reported factors impeding non-western women’s use of prenatal care.

**Table 1:** **Overview of the factors according to the conceptual framework of Foets *et al.***

	**Category**	**Barriers**	**Facilitators**
Individual factors	**Demographics, genetics and pregnancy**	Being younger than 20 [20]*	
		Multiparity [20]*	
		Unplanned pregnancy [20]*	
	**Migration**	Lack of knowledge of or information about the Western healthcare system [22,23,25-27,30-32,35]	Recognition of prenatal care as an important issue in the community [30]^•^
		Arriving in the new country late in pregnancy [22]*	
	**Culture**	Adherence to cultural and religious practices [23,25,34]^•^	Care provider of the same ethnic origin [27]^•^
		Poor language proficiency [20,22,24,26,27,30,31]	Belief that prenatal care ensures baby’s well-being [23,34]^•^
		Lack of assertiveness [24]^•^	Belief in looking after your own health for a healthy baby [34]^•^
		Dependency on husband [22,34,35]	
		Perceiving pregnancy as a normal state [29]^•^	
		Belief that prenatal care is more a burden than a benefit [25]^•^	
		Belief that prenatal classes are not necessary [22,34]	
	**Position in host country**	Financial problems [22,23,31]	Better socio-economic follow-up [31]^•^
		Unemployment [21]*	
		Low or intermediate educational level [20,21]*	
		Social inequality (education, economic resources and residence (rural or urban)) [35]^•^	
		Lack of time [22,23,27,30]	
		Lack of childcare [23,25]^•^	
		No medical leave from work [31]^•^	
	**Social network**	No support from family [35]^•^	Husband with a good command of the industrialized country’s official language [34]^•^
		Acquiring or following advice from family and friends [22,23]	
		Isolated community [35]^•^	
Health service factors	**Accessibility**	Inappropriate timing and incompatible opening hours [23,35]^•^	
		Transport and mobility problems [22,26,27,35]	
		Indirect discrimination [32]^•^	
	**Expertise**	Care provider lacking knowledge of cultural practices [25]^•^	A mature, experienced healthcare provider with a command of the native language [30]^•^
			Care provider showing interest and respect [23]^•^
			Care provider alleviating worries and fears [23]^•^
	**Personal treatment and communication**	Poor communication [23,32,35]^•^	Use of native language [27,28,30,31]^•^
		Perceiving yourself as having been badly treated by a care provider [33]^•^	Improved communication [23,31]^•^
			Audio-visual material [27]^•^
			Renaming prenatal classes to prenatal sessions [30]^•^

Ad* Factors only reported in quantitative studies.

Ad• Factors only reported in qualitative studies or the qualitative part of the mixed-methods study.

Demographic, genetic and pregnancy-related factors were only described in one quantitative study and in none of the qualitative studies. In this study *multiparity, being younger than 20* and *unplanned pregnancy* were associated with late prenatal care entry [20].

On the other hand, expertise factors as well as personal treatment and communication factors were only described in qualitative studies. *Care providers with a lack of knowledge of cultural practices* were described as being unable to provide knowledgeable health guidance and more likely to display insensitive behaviour [25]. Interviews with caregivers revealed that Somali women p*erceiving themselves as being treated badly by a care provider* would not return for antenatal care [33]. *Poor communication* complicated women’s access to prenatal care [35], prevented attendance of prenatal classes [23] and was reported as an underlying problem in understanding maternity reproductive services [32].

Factors reported in both qualitative and quantitative studies concerned: migration, culture, position in the host country, social network and accessibility of prenatal care.

Migration-related factors: For Asian, Somali and Turkish women, as well as Muslim women otherwise unspecified, *lack of knowledge of or information about the Western healthcare system* was reported to deter utilization of prenatal care [26,27,30-32,35] or prenatal classes [22,23,25]. *Arriving in the new country late in pregnancy* was reported as another reason for not attending prenatal classes [22].

Cultural factors: *Adherence to cultural and religious practices* was reported to impede prenatal care utilization by Asian and Muslim women. Women entered prenatal care late because of shame about being undressed during consultations [23]. Prenatal classes were not attended because of feelings of fear and embarrassment about watching a video of the act of giving birth [34] and because classes were not exclusively designed for women [25]. *Poor language proficiency* was another cultural characteristic described as an impeding factor for prenatal care [20,22,24,27,30,31] and prenatal classes [26]. *Lack of assertiveness* appeared to make it difficult for Asian women to access maternity services and information. These women were too reluctant or ashamed to enquire about services or ask for information [24]. *Dependency on the husband* was described as complicating access to both prenatal care [35] and prenatal classes [22,34]. *Pregnancy was perceived as a normal state* by Somali women and some of them therefore did not understand the necessity of prenatal care [29]. *Prenatal care was perceived as a burden more than a benefit* because the same procedure is performed every time and doctors are too busy to provide pregnancy-related information [25]. *Prenatal classes were perceived as not being necessary* as women had already experienced birth [22,34] or attended classes previously [22].

Factors related to women’s position in the host country: *Financial problems* impeded the ability to pay for health insurance [31], access to medical care during pregnancy [22] and attendance of prenatal classes [23]. *Unemployment* was another characteristic that was identified. In a Dutch study, enabling factors (including being in employment) explained Turkish women’s delayed entry into prenatal care [21]. In two studies, *low or intermediate educational level* was associated with late entry into prenatal care [20,21]. *Social inequalities* in education, economic resources and residence (rural or urban) among those who have immigrated, were found to affect access to prenatal care [35]. *Lack of time* was reported as a reason for not attending prenatal classes [22,23,30] and as a barrier to accessing prenatal support from public health and community nurses [27]. Another reason for not attending prenatal classes was *lack of childcare*[23,25]. Turkish women in a Swiss study reported *problems obtaining medical leave from work*[31].

Social network factors: *Little or no support from family* was described as complicating access to prenatal care [35]. *Acquiring or following advice from family and friends* was reported as a reason for not attending prenatal classes [22,23]. *Isolation of the community* was described as complicating Chinese women’s access to prenatal care [35].

Accessibility factors: *Inappropriate timing* was reported as a reason for not attending prenatal classes [23] while *incompatible opening hours* (incompatible with women’s own working hours or those of their husband or accompanying persons) were reported to affect their access to prenatal care [35]. *Transport and mobility problems* were reported to complicate access to medical care during pregnancy [22], prenatal care [35] and prenatal classes [26,27]. *Indirect discrimination* also affected access to care. Somali women in a UK study reported that general practitioners would sometimes refuse to see them if they did not bring along an interpreter, and that they had to book appointments for secondary care three days in advance if interpretation services were needed [32].

### Facilitators of prenatal care utilization

All factors facilitating the use of prenatal care were classified as barriers. The second column of Table 1 gives an overview of these factors according to the conceptual framework of Foets *et al.*. These factors were only reported in qualitative studies and concerned: migration, culture, socioeconomic status, social network, treatment and communication.

Migration-related factors: To improve prenatal class attendance, women suggested *recognition of prenatal care as an important issue in the community* through mobilisation within their communities by word of mouth, radio and television [30].

Cultural factors: Women felt that prenatal support provided by *health workers or peers of the same ethnic origin* would be beneficial to them [27]. *Believing that prenatal care ensures babies’ well-being* was another characteristic that facilitated prenatal care utilization. In one study, prenatal care was perceived as an important aspect of pregnancy that could assure women about their babies’ well-being [34], while in another study regular consultations reduced women’s uncertainty or fear about the pregnancy or their babies’ health [23]. *Believing in looking after your own health for a healthy baby* was also described as a reason for not missing any prenatal check-ups [34].

Factors related to women’s position in the host country: Women suggested *better socioeconomic follow-up by institutions* because socioeconomic conditions affected their ability to pay for health insurance [31].

Social network factors: Women with a *husband who spoke the industrialized country’s official language* reported that their husbands told them to attend antenatal check-ups and arranged antenatal care because they did not speak the country’s language themselves [34].

Expertise factors: Women recommended that *healthcare providers facilitating prenatal care sessions should be mature women with experience of childbirth*[30]. Care providers were expected to *show respect by being interested and allowing for women’s sense of shame about nudity*[23]. They were also expected to *alleviate worries and fears* by giving women a sense of security through careful monitoring, assessment, supervising and by acknowledging women’s fears and reassuring them [23].

Personal treatment and communication factors: One of these factors was the *use of women’s native language.* Women proposed more information in their native language [31], prenatal classes being conducted in their native language [27] and healthcare providers with a command of their native language [30]. Group prenatal care was described as being more accessible when practice midwives spoke several community languages [28]. Another characteristic was *improved communication.* Care providers or institutions were expected to provide translation [23,31], conversation space [23], and to make up for women’s experience and knowledge by asking specific questions and giving customized information, demonstrations and explanations [23]. In one study, women reported a *preference for audio-visual material* over written information [27]. Women explained that the term “classes” suggests that they are ignorant about childbirth, and that *prenatal classes should be called prenatal sessions* to improve their attendance [30].

## Discussion

### Factors affecting prenatal care utilization

This review gives an overview of factors affecting non-western women’s use of prenatal care in western societies. Therefore, ‘factors’ were described in the broadest sense, comprising experiences, needs and expectations, circumstances, characteristics and health beliefs of non-western women. The results indicate that non-western women’s use of prenatal care is influenced by a variety of factors, and that several factors may simultaneously exert their effect. The categories migration, culture, position in the host country, social network, expertise of the care provider and personal treatment and communication were found to include both facilitating and impeding factors for non-western women’s prenatal care utilization. The category demographics, genetics and pregnancy and the category accessibility of care only included impeding factors. The only aspect of the conceptual framework of Foets *et al.* that was not found in the studies included in this review was ‘professionally defined need’.

In a systematic review conducted by Feijen-de Jong *et al.*, ethnic minority was found to be one of the determinants of inadequate prenatal care utilization in high income countries [16]. As ethnic minority status does of itself not explain prenatal care utilization, our review adds relevant information to the review by Feijen-de Jong and colleagues, and gives more insight into the factors behind these women’s prenatal care utilization, at least for those of non-western origin. The demographic and socioeconomic factors found in our review are largely in line with the results of Feijen-de Jong *et al.*. However, we did not find any factors concerning pattern or type of prenatal care, planned place of birth, prior birth outcomes and health behaviour. Our results are also in line with the review by Heaman *et al.*, who reported that demographic, socioeconomic and language factors affected prenatal care utilization by first generation migrant women [17]. In addition to these two reviews, we found several other factors at the individual and health service levels that impeded or facilitated non-western women’s prenatal care utilization.

To our knowledge, this is the first review of prenatal care utilization by non-western women that has combined quantitative, qualitative and mixed-methods studies. By doing this, we were able to find a very wide range of factors affecting non-western women’s prenatal care utilization. This is clearly evident from the barriers. A comparison shows that the quantitative studies made a full contribution to inadequate users’ demographic, genetic and pregnancy characteristics. All three factors in this category: namely being younger than 20, multiparity and unplanned pregnancy were derived from one quantitative study. The qualitative studies contributed fully to expertise factors as well as personal treatment and communication factors. Care providers lacking knowledge of cultural practices, poor communication and perceiving yourself as having been badly treated by a care providers were only derived from qualitative studies and the qualitative part of the mixed methods study. Besides providing all the barriers in a specific category, quantitative and qualitative studies also complemented each other by both providing barriers in the same category (migration, culture, position in the host country, social network, accessibility), sometimes even by means of the same barrier. The factors: lack of knowledge of or information about the Western healthcare system, poor language proficiency, dependency on husband, belief that prenatal care is not necessary, financial problems, lack of time, acquiring or following advice from family and friends, and transport and mobility factors were all reported in quantitative as well as qualitative studies.

By combining different study designs, we were also able to provide more in-depth insight into the mechanisms of some factors. For instance, we obtained more insight into the mechanisms of the factor multiparity reported in two previous quantitative studies. Qualitative studies showed that multiparous women did not perceive prenatal classes as necessary because they had already given birth. Furthermore, multiparous women reported lack of childcare as a reason for not attending prenatal classes. Perhaps these two reasons also play a role in multiparous women’s utilization of medical care during pregnancy.

In the introduction, non-western women’s risk for adverse pregnancy outcomes was described according to region of origin. By placing this review’s findings in a regional perspective, some noteworthy insights were gained about factors affecting these high risk groups’ health care utilization. As to individual barriers, lack of knowledge of the Western healthcare system was described among all four regional groups distinguished in this review (sub-Saharan African, North African, Asian and Turkish). Health beliefs were reported among sub-Saharan African (Somali) and Asian women. Dependency on husband was reported among Asian and North African women. However, adherence to cultural practices, acquiring or following advice from family and friends, lack of assertiveness and lack of time were only described in studies conducted among Asian women. As to health service barriers, accessibility factors were reported in studies conducted among Asian and North African woman. On the other hand, expertise and personal treatment factors were only found among sub-Saharan African (Somali) women.

These insights can be used to develop a more targeted approach towards specific groups. For example by placing emphasis on ‘dependency on husband’ for Asian and North African women, and ‘personal treatment’ for sub Saharan women. However, this should be done carefully. Some factors may seem to play no role for certain ethnic groups, while they were simply not included or discussed in these studies.

The individual and health service facilitators were all derived from qualitative studies conducted among Asian women and Turkish women. Nevertheless, these facilitating factors can be applicable to other ethnic groups, as they relate to difficulties also reported by these groups (e.g. improved communication).

Several factors such as lack of knowledge or information of the western healthcare system, poor language proficiency and poor communication applied to women of various ethnic origins. On the other hand, some factors were highly specific to a country, culture or religion. Muslim women, for example, were found to refuse combined session with males while other women might have fewer gender issues. Extrapolation of the results is therefore less applicable. The factors reported to facilitate prenatal care utilization were mostly suggestions made by women. As women based these suggestions on their own experiences with prenatal care, we decided to include these in our review.

In a systematic review conducted by Simkahada et al., perceiving pregnancy as a normal state and seeing little direct benefit from antenatal care were reported as barriers to antenatal care utilization in developing countries [36]. In our review, we found somewhat similar impeding beliefs about prenatal care in two studies conducted among first generation women. Furthermore, Simkhada and colleagues reported unsupportive family and friends as a barrier to antenatal care utilization which was also found in our review. These similarities between non-western women in industrialized western countries and women in developing countries indicate that some women seem to continue to have certain beliefs, attitudes and needs they had prior to migration. A comparison between first and second generation non-western women would be very useful, but was not possible. Only one study included second generation women but presented the results in combination with first generation women.

Even though we included only high-income countries with universally accessible healthcare, we found that financial factors did affect non-western women’s prenatal care utilization. One explanation for this finding might be that women may not be aware of the universal accessibility of care, and therefore perceive lack of money as a barrier to prenatal care. It might also be that, even though women are currently legally resident (which was an inclusion criterion of our review), they reflect back on periods when this was not the case.

### Methodological reflections

One noteworthy point is the large number of qualitative studies included in this review, as compared to quantitative studies. During the review process, we identified several quantitative studies focusing on factors affecting prenatal care utilization by non-western women among their study population. Regrettably, we had to exclude most of these studies as they lacked a sub-analysis specifically for non-western women. By doing a sub-analysis specifically for non-western women in future quantitative studies on prenatal care utilization, more insights can be gained on factors affecting their use of prenatal care.

The studies included in this review all considered different subgroups of non-western women. However, the immigrant generation of the women was not reported in five studies and factors were not specified according to generation in the only study that included first and second generation women.

The factors found in the qualitative studies were mostly part of women’s experiences, needs and expectations with prenatal care. These studies did not specifically focus on inadequate users, and therefore did not include a definition. On the contrary, two of the three quantitative studies defined inadequate use, but did so differently (Additional file 3). This difference in definition between the quantitative studies and the lack of definition in qualitative studies complicates comparison and integration of the study results.

The included studies showed a large variance in methodological quality. Nevertheless, we decided not to exclude studies with a low quality score, in order to prevent loss of any relevant factors in this review. Instead we compared the results of the high and low methodological quality studies against each other, and did not find any contradictory results.

Two main strengths of this study are the use of a broad search string and not applying a language restriction, to minimize the chance of missing relevant studies. Also the inclusion of quantitative, qualitative and mixed-methods studies adds to the strength, as this increases the chance of finding different types of relevant factors affecting prenatal care utilization. Another strength is the restriction to countries with universally accessible healthcare. Therefore, results are more comparable and generalizable to other countries with a similar organization of their healthcare system. The use of a theoretical framework to sort the factors found is another strength of the study, as this gives a clear overview of the factors and the level at which they exert their effect.

## Conclusions

Sixteen studies heterogeneous in methodological quality were included in this review. A variety of factors at the individual and health service levels were found to affect non-western women’s use of prenatal care. Lack of knowledge of the western healthcare system and poor language proficiency were the most frequently reported impeding factors, while provision of information and care in women’s native language was the most frequently reported facilitating factor. The factors found could all be classified according to the conceptual framework of Foets *et al.*, and covered all categories with the exception of ‘professionally defined need’.

The factors reported were mainly derived from qualitative studies, and more detailed quantitative research with sub-analyses for non-western women is needed to determine the magnitude of these factors’ effects on prenatal care utilization. Furthermore, more qualitative studies specifically aimed at non-western women making inadequate use of prenatal care are necessary.

The factors found in this review provide specific indications for identifying non-western women at risk of inadequate use of prenatal care, and developing interventions and adequate policy aiming at improving their prenatal care utilization.

## Competing interests

The authors declare that they have no competing interests.

## Authors’ contributions

All authors have made substantial contributions to this study. AB and WD developed the review with the support of TW, JM and AF. AB conducted the search, and all authors contributed to the screening, data extraction and quality assessment. The final version of the manuscript was read and approved by all authors.

## Pre-publication history

The pre-publication history for this paper can be accessed here:

http://www.biomedcentral.com/1471-2393/13/81/prepub

## Supplementary Material

Additional file 1 Search strategy in PubMed. Click here for file

Additional file 2 Overview of the study characteristics. Click here for file

Additional file 3 Additional information of the included studies. Click here for file
