# Supplementary material for: Factors affecting the use of prenatal care by non-western women in industrialized western countries: a systematic review
Source: BMC Pregnancy Childbirth. 2013 Mar 27;13:81. doi: 10.1186/1471-2393-13-81 (PMC3626532; doi:10.1186/1471-2393-13-81)
Supplement: Additional file 1 — Search strategy in PubMed. [file 1471-2393-13-81-S1.doc]

**Search strategy in PubMed**

The strategy used in PubMed is given below. This strategy consists of four strings and was adapted for use in the other databases.

# 1

**Search ((((((((((prenatal care) OR antenatal care) OR pregnancy care) OR midwifery care) OR postnatal care) OR postpartum care) OR maternity nursing) OR maternal child nursing) OR maternal-child nursing) OR maternal healthcare) OR maternal health care**

(((((((((("prenatal care"[MeSH Terms] OR ("prenatal"[All Fields] AND "care"[All Fields]) OR "prenatal care"[All Fields]) OR ("prenatal care"[MeSH Terms] OR ("prenatal"[All Fields] AND "care"[All Fields]) OR "prenatal care"[All Fields] OR ("antenatal"[All Fields] AND "care"[All Fields]) OR "antenatal care"[All Fields])) OR ("prenatal care"[MeSH Terms] OR ("prenatal"[All Fields] AND "care"[All Fields]) OR "prenatal care"[All Fields] OR ("pregnancy"[All Fields] AND "care"[All Fields]) OR "pregnancy care"[All Fields])) OR (("midwifery"[MeSH Terms] OR "midwifery"[All Fields]) AND care[All Fields])) OR ("postnatal care"[MeSH Terms] OR ("postnatal"[All Fields] AND "care"[All Fields]) OR "postnatal care"[All Fields])) OR ("postnatal care"[MeSH Terms] OR ("postnatal"[All Fields] AND "care"[All Fields]) OR "postnatal care"[All Fields] OR ("postpartum"[All Fields] AND "care"[All Fields]) OR "postpartum care"[All Fields])) OR ("maternal-child nursing"[MeSH Terms] OR ("maternal-child"[All Fields] AND "nursing"[All Fields]) OR "maternal-child nursing"[All Fields] OR ("maternity"[All Fields] AND "nursing"[All Fields]) OR "maternity nursing"[All Fields])) OR ("maternal-child nursing"[MeSH Terms] OR ("maternal-child"[All Fields] AND "nursing"[All Fields]) OR "maternal-child nursing"[All Fields] OR ("maternal"[All Fields] AND "child"[All Fields] AND "nursing"[All Fields]) OR "maternal child nursing"[All Fields])) OR ("maternal-child nursing"[MeSH Terms] OR ("maternal-child"[All Fields] AND "nursing"[All Fields]) OR "maternal-child nursing"[All Fields] OR ("maternal"[All Fields] AND "child"[All Fields] AND "nursing"[All Fields]) OR "maternal child nursing"[All Fields])) OR (("mothers"[MeSH Terms] OR "mothers"[All Fields] OR "maternal"[All Fields]) AND ("delivery of health care"[MeSH Terms] OR ("delivery"[All Fields] AND "health"[All Fields] AND "care"[All Fields]) OR "delivery of health care"[All Fields] OR "healthcare"[All Fields]))) OR ("maternal health services"[MeSH Terms] OR ("maternal"[All Fields] AND "health"[All Fields] AND "services"[All Fields]) OR "maternal health services"[All Fields] OR ("maternal"[All Fields] AND "health"[All Fields] AND "care"[All Fields]) OR "maternal health care"[All Fields])

#2

**Search ((((((((ethnicity) OR ethnic group) OR ethnic groups) OR ethnic minority) OR ethnic minorities) OR immigrants) OR migrants) OR foreigners) OR foreign nationals**

(((((((("ethnology"[Subheading] OR "ethnology"[All Fields] OR "ethnicity"[All Fields] OR "ethnology"[MeSH Terms] OR "ethnicity"[All Fields] OR "ethnic groups"[MeSH Terms] OR ("ethnic"[All Fields] AND "groups"[All Fields]) OR "ethnic groups"[All Fields]) OR ("ethnic groups"[MeSH Terms] OR ("ethnic"[All Fields] AND "groups"[All Fields]) OR "ethnic groups"[All Fields] OR ("ethnic"[All Fields] AND "group"[All Fields]) OR "ethnic group"[All Fields])) OR ("ethnic groups"[MeSH Terms] OR ("ethnic"[All Fields] AND "groups"[All Fields]) OR "ethnic groups"[All Fields])) OR (("ethnic groups"[MeSH Terms] OR ("ethnic"[All Fields] AND "groups"[All Fields]) OR "ethnic groups"[All Fields] OR "ethnic"[All Fields]) AND ("minority groups"[MeSH Terms] OR ("minority"[All Fields] AND "groups"[All Fields]) OR "minority groups"[All Fields] OR "minority"[All Fields]))) OR (("ethnic groups"[MeSH Terms] OR ("ethnic"[All Fields] AND "groups"[All Fields]) OR "ethnic groups"[All Fields] OR "ethnic"[All Fields]) AND ("minority groups"[MeSH Terms] OR ("minority"[All Fields] AND "groups"[All Fields]) OR "minority groups"[All Fields] OR "minorities"[All Fields]))) OR ("emigrants and immigrants"[MeSH Terms] OR ("emigrants"[All Fields] AND "immigrants"[All Fields]) OR "emigrants and immigrants"[All Fields] OR "immigrants"[All Fields])) OR ("transients and migrants"[MeSH Terms] OR ("transients"[All Fields] AND "migrants"[All Fields]) OR "transients and migrants"[All Fields] OR "migrants"[All Fields])) OR ("emigrants and immigrants"[MeSH Terms] OR ("emigrants"[All Fields] AND "immigrants"[All Fields]) OR "emigrants and immigrants"[All Fields] OR "foreigners"[All Fields])) OR (("internationality"[MeSH Terms] OR "internationality"[All Fields] OR "foreign"[All Fields]) AND nationals[All Fields])

#3

(#2) AND #1

#4

(#2) AND #1 AND ("1995/01/01"[PDAT] : "2012/07/05"[PDAT])
