# Supplementary material for: Factors affecting the use of prenatal care by non-western women in industrialized western countries: a systematic review
Source: BMC Pregnancy Childbirth. 2013 Mar 27;13:81. doi: 10.1186/1471-2393-13-81 (PMC3626532; doi:10.1186/1471-2393-13-81)
Supplement: Additional file 2 — Overview of the study characteristics. [file 1471-2393-13-81-S2.doc]

**Overview of the study characteristics**

| **Authors** | **Goal/aim** | **Study design** | **Sample size & ethnic group** | **Non-western women** | | | **Methodological quality score** |
| --- | --- | --- | --- | --- | --- | --- | --- |
|  |  |  |  | **Generation** | **Age** | **Parity** |  |
| # 20: Alderliesten et al. 2007 (the Netherlands (Amsterdam)) | To investigate the difference in timing of the first antenatal visit between ethnic groups and to explore the contribution of several noneconomic risk factors | Quantitative: prospective cohort study | 8238 women: - Surinamese: 458 - Antillean: 95 - Turkish: 342 - Moroccan: 604 - Ghanaian: 203 - Other non-western: 767 - Dutch: 5071  - Other western: 698 | Not mentioned | <20 years:  - Surinamese: 3.1% - Antillean: 14.7%  - Turkish: 5.3%  - Moroccan: 3.6%  - Ghanaian: 2.0%  - Other non-western: 3.8% | Multiparity: - Surinamese: 60.1%  - Antillean: 38.9%  - Turkish: 63.2%  - Moroccan: 59.9%  - Ghanaian: 62.6%  - Other non-western: 51.8% | 100% |
| # 21: Choté et al. 2011 (the Netherlands (Rotterdam)) | To examine whether and to what extent ethnic differences between Dutch and several non-Dutch groups in late entry into antenatal care by community midwives can be explained by need, predisposing and enabling factors | Quantitative: prospective cohort study | 2093 women: - Turkish: 240 - Moroccan: 208 - Surinamese Creole: 76 - Surinamese Hindustani: 86 - Antillean: 108 - Cape Verdean: 133 - Dutch: 1242 | First and second generation | Mean age/Standard deviation: -Turkish: 25.7 (4.4)  - Moroccan: 27.7 (4.9)  - Cape Verdean: 26.7 (5.7)  - Antillean: 25.7 (4.7)  - Surinamese Creole: 26.9 (6.1)  - Surinamese Hindustani: 26.4 (4.9) | ≥1 child (%):  - Turkish: 46.7%  - Moroccan: 65.9%  - Cape Verdean: 36.8%  - Antillean: 38.9%  - Surinamese Creole: 40.8 %  - Surinamese Hindustani: 40.7% | 75% |
| # 22: Brar et al. 2009 (Canada (Calgary, Alberta)) | To assess the use of perinatal services by newly immigrated South Asian women and Canadian-born women, and to determine any perceived barriers to receiving care | Quantitative: matched-sample, stratified, cross-sectional, descriptive telephone survey | 30 South Asian women: - Indian: 16 - Pakistani: 13 - Bangladeshi: 1 &  30 Canadian born women of any ethnicity | First generation | Mean age: 25.8 years | Median: 1.5 children | 75% |
| # 23: Büchi et al. 2006 (Switzerland) | To study the needs and expectations of Sri Lankan Tamil women to promote their satisfaction and optimal use of prenatal care | Qualitative: problem centred interviews before and after delivery | 7 Tamil women of Sri Lanka | First generation | Age range: 22-33 years | 1 child: 3 women  2 children: 3 women 4 children: 1 woman | 50% |
| # 24: Hoang et al. 2009 (Australia (Tasmania)) | To investigate Asian migrant women’s childbirth experiences in a rural Australian context | Qualitative: semi-structured interviews | 10 Asian women: - Vietnamese: 4 - Chinese: 2 - Japanese: 2 - Korean: 1 - Filipino: 1 | First generation | Not reported | All 10 women had one to two children born in Australia | 50% |
| # 25: Reitmanova and Gustafson 2008 (Canada (St. John's)) | To document and explore the maternity healthcare needs and the barriers to accessing maternity health services from the perspective of immigrant Muslim women living in St. John's, Canada | Qualitative: in-depth semi-structured interviews | 6 Muslim women | First generation | Age range: 25-40 years s | These 6 women had a total of 18 children | 75% |
| # 26: Stewart et al. 1998 (Australia (Brisbane)) | To understand and explore the notion of empowerment in relation to the childbirth experiences of Filipino women in Brisbane, Australia | Qualitative: in-depth face to face interviews | 30 Filipino women | First generation | Not reported | Not reported | 50% |
| # 27: Sutton et al. 2007 (Canada (London, Ontario)) | Exploring Vietnamese women's breastfeeding experience, and their families' needs for prenatal and postpartum health professional programs and services | Qualitative: in-depth interviews | 11 Vietnamese women | Mostly first generation women | 21 years: 1 woman 24 years: 2 women 25 years: 1 woman 31 years: 1 woman 34 years: 1 woman 35 years: 1 woman 37 years: 1 woman 38 years: 1 woman 39 years: 1 woman 42 years: 1 woman | 1 child: 5 women 2 children: 3 women 4 children: 1 woman 6 children: 2 women | 50% |
| # 28: McAree et al. 2010 (United Kingdom) | To explore women's perceptions of care with a midwifery group practice compared to experiences of standard maternity care in an ethnically diverse area | Qualitative: individual semi-structured interviews | 18 Women: - Indian: 7 - Sri Lankan: 1 - Pakistani: 2 - African/African British: 1 - Black Caribbean/Black: 1 - White British/European: 6 | Not reported | Not reported | Not reported | 75% |
| # 29: Essén et al. 2000 (Sweden) | To explore the culturally determined attitudes, strategies and habits of Somali immigrant women towards pregnancy and childbirth in Somalia as well as Sweden, in order to gain an understanding of how such factors affect perinatal outcome | Qualitative: interpreter assisted in-depth interviews | 15 Somali women | First generation | Age range: 20-55 years | Range: 2-9 children | 75% |
| # 30: Bhagat et al. 2002 (Canada (Lower mainland of British Columbia)) | To explore how community mobilization strategies could be used to improve the health of pregnant women in the Punjabi community | Qualitative: focus groups | Punjabi (Indian) women.  Sample size not reported | Not reported | Not reported | Not reported | 25%* |
| # 31: Bollini et al. 2007 (Switzerland (La-Chaux-de-Fonds; Bern; Zurich; Fribourg)) | To explore the issues of pregnancy and delivery in migrant women in their interaction with the Swiss healthcare system | Qualitative:  focus groups | 40 women: - Turkish: 14 - Portuguese: 17 - Swiss: 9 | First generation | <30 years: 2 women 31-50 years: 9 women  >50 years: 3 women | 1 child: 3 women  2 children: 5 women  >2 children: 6 women | 75% |
| # 32: Davies and Bath 2001 (the United Kingdom (in a northern English city)) | To explore the maternity information concerns of a group of Somali women in a Northern English city and to investigate the relationships of these women with maternity health professionals | Qualitative: a focus group and semi-structured interviews | 13 Somali women | Not reported | 21-30 years : 6 women 31-40 years: 7 women | The 6 women between 21 and 30 had borne a total of 18 children. The 7 women between 31 and 40 had borne a total of 22 children. | 75% |
| # 33: Binder et al. 2012 (United Kingdom (Greater London)) | To gain a deeper understanding of the multi-ethnic care setting and the roles that ethnicity and language play during the sensitive care encounter between immigrant women and their western care providers | Qualitative: in-depth individual and focus group interviews | 60 Women: - Somali: 39 - Ghanaian: 11 - White British: 10 & 62 Obstetric care providers: - Somali: 4 - Other African or Caribbean: 34 - Asian: 3 - White British: 21 | First generation | Age range: 18-48 years | Range: 1-10 children | 100% |
| # 34: Rice and Naksook 1998 (Australia (Melbourne)) | To identify the perceptions and experience of pregnancy care, labour and birth of Thai women in Melbourne, Australia | Qualitative: ethnographic interviews and participant observation | 30 Thai women | First generation | 20-30 years: 9 women  31-40 years: 15 women  41-50 years: 4 women  50+ years: 2 women | 1 child: 13 women  2 or 3 children: 15 women  4-6 children: 2 women | 50% |
| # 35: Baken et al. 2007 (Italy (Cesena area))  Only the qualitative part is included in this review! | To study the health needs, the demand for inpatient care and access to health services by immigrant children and women in the district Cesena, with the purpose to gain useful insights to improve service organisation and program interventions focused specifically on the maternal and infant population | Mixed methods: - Quantitative: cohort study - Qualitative: separate focus groups with immigrants, cultural mediators and health professionals | Qualitative part: 103 persons: Chinese women, Northwest African women (Maghreb), other immigrant women otherwise unspecified, cultural mediators and health professionals | Not reported | Not reported | Not reported | 25% |

Ad* Partially met the first and second criterion
