# Supplementary material for: Factors affecting the use of prenatal care by non-western women in industrialized western countries: a systematic review
Source: BMC Pregnancy Childbirth. 2013 Mar 27;13:81. doi: 10.1186/1471-2393-13-81 (PMC3626532; doi:10.1186/1471-2393-13-81)
Supplement: Additional file 3 — Additional information of the included studies. [file 1471-2393-13-81-S3.doc]

**Additional information of the included studies**

| **Authors** | **Definition for adequacy of prenatal care utilization** | **Other characteristics of the non-western women** | **Barriers** | **Facilitators** |
| --- | --- | --- | --- | --- |
| # 20: Alderliesten et al. 2007 (the Netherlands (Amsterdam)) | Late start: gestational age at first visit ≥18 weeks Very late start: gestational age at first visit ≥24 weeks | Unplanned pregnancy Surinamese: 15.3% Antillean: 12.9% Turkish: 19.4% Moroccan: 8.9% Ghanaian: 14.1% Other non-western: 12.1%  Pregnancy unwanted Surinamese: 1.1% Antillean: 1.1%  Turkish: 5.0% Moroccan: 2.5% Ghanaian: 2.0% Other non-western: 1.7%  Low maternal education Surinamese: 43.0%  Antillean: 42.6%  Turkish: 64.3%  Moroccan: 60.9%  Ghanaian: 56.1%  Other non-western: 39.5%  Poor language proficiency in Dutch Surinamese: 1.1% Antillean: 1.1%  Turkish: 43.1% Moroccan: 34.2% Ghanaian: 46.3% Other non-western: 37.9%  Risk awareness Surinamese: 25.3%  Antillean: 26.3% Turkish: 22.2%  Moroccan 24.1% Ghanaian 31.0% Other non-western: 21.9% | Turkish, Moroccan, Ghanaian, Other non-western women:  1) Less than 5 years education after primary school 2) Unplanned pregnancy 3) Being younger than 20 4) Multiparity 5) Poor Dutch language proficiency | Not reported |
| # 21: Choté et al. 2011 (the Netherlands (Rotterdam)) | Late entry: gestational age at first visit more than 14 weeks | Pregnancy planned:  Turkish: 55.8%  Moroccan: 60.6% Cape Verdean: 37.6%  Antillean: 30.6%  Surinamese Creole: 40.8%  Surinamese Hindustani: 44.2%  Paid job:  Turkish: 28.3%  Moroccan: 19.2% Cape Verdean: 42.9%  Antillean: 19.4%  Surinamese Creole: 39.5%  Surinamese Hindustani: 30.2%  Married or cohabiting:  Turkish: 90.1%  Moroccan: 95.7% Cape Verdean: 44.3%  Antillean: 45.3%  Surinamese Creole: 42.1%  Surinamese Hindustani: 73.3%  Higher educational level:  Turkish: 12.1%  Moroccan: 12.0% Cape Verdean: 9.0%  Antillean: 12.0%  Surinamese Creole: 15.8%  Surinamese Hindustani: 10.5% | Turkish women: 1) Enabling factors : low or intermediate educational level, not having a paid job | Not reported |
| # 22: Brar et al. 2009 (Canada (Calgary, Alberta)) | Not applicable | Recently delivered a live born term healthy singleton in an uncomplicated vaginal delivery at the Peter Lougheed Centre, an academic community hospital in NE Calgary  Speaking Hindi, Punjabi and/or English  Having a permanent address  Having access to a telephone  Intending to parent | South Asian women (Indian, Pakistani, Bangladeshi):  1) Language barriers  2) Transport or mobility problems  3) Lack of money  4) Not advised to attend prenatal education classes  5) Unaware of prenatal education classes  6) Arriving in Canada late in pregnancy 7) Acquiring prenatal knowledge from family and friends 8) Husband unwilling to attend prenatal classes 9) Having attended classes previously 10) Having previous children 11) Lack of time | Not reported |
| # 23: Büchi et al. 2006 (Switzerland) | Not applicable | All women were married  The women lived between 1 and 16 years in Switzerland  The women’s education ranged from eight to ten years. They did not finish their education, except for one (secretary) | Tamil (Sri Lanka) women:  1) Shame about being undressed during consultations 2) Limited communication 3) Financial problems 4) Inappropriate timing 5) Lack of time 6) Lack of childcare 7) Unfamiliarity with prenatal classes 8) Prenatal classes not recommended by Tamil friends | Tamil (Sri Lanka) women:  1) Reducing uncertainty or fear about the child's health or pregnancy through regular consultations 2) Care providers should show respect by being interested and allowing for women’s sense of shame about nudity 3) Care providers should alleviate worries and fears by giving a sense of security through careful monitoring, assessment and supervising, and by acknowledging women’s fears and reassuring them 4) Care providers should make up for women's lack of experience and knowledge by asking specific questions and by giving customized information, demonstrations and explanations  5) Care providers should ensure communication by providing translation and creating conversation space |
| # 24: Hoang et al. 2009 (Australia (Tasmania)) | Not applicable | Two women spoke limited English, the other women could communicate in English | Asian women (Vietnamese, Chinese, Japanese, Korean, Filipino):  1) Lack of English language skills 2) Too reluctant or embarrassed to express their needs or enquire about services, because in most Asian cultures people are taught to be unassertive and inhibited | Not reported |
| # 25: Reitmanova and Gustafson 2008 (Canada (St. John's)) | Not applicable | All participants considered themselves believing Muslim's, however the level of their religious practices varied  All participants were married to men of the same ethnic and religious background  The participants socioeconomic status varied depending on their husband's employment circumstances  All participants were stay-at-home mothers, while they all had a high school diploma or university degree | Muslim women:  1) Prenatal check-ups are considered to be a routine that does not provide any important benefits. In some instances they are perceived to be more as a burden than benefit, as the same procedure is performed every time and doctors are too busy and don't have much time to provide pregnancy related information. 2) Not being told about or not understanding the purpose of prenatal classes 3) No care arrangements for other children 4) Prenatal classes were not designed exclusively for women which contravenes to their religious beliefs 5) Maternity care providers were uninformed about religious practices, and thus unable to provide knowledgeable health guidance taking these religious needs into consideration and also more likely to display insensitive behaviour | Not reported |
| # 26: Stewart et al.1998 (Australia (Brisbane)) | Not applicable | Post-partal women, who had experienced their first birth in Brisbane  Resident in Australia: more than 4 years (20); less than 4 years (10)  English language capacity (speak, read, write): good (26); poor (4)  Educational level (completed): secondary (15); tertiary (14)  Employment: paid employment (26); domestic duties (4)  Occupational status: white collar (8); blue collar (18)  Partner employment: employed (16); aged pensioner (14)  Personal income: up to $16000 (18); $16001-$23000 (8)  Family income per month: $1000-2000 approx.(27); $2000 or more (3)  Partner origins: white Australian (20); Filipino (10) | Filipino women:  1) Language barriers 2) Not familiar with/informed about additional services such as antenatal care (shared care or midwives clinic) 3) Public transport | Not reported |
| # 27: Sutton et al. 2007 (Canada (London, Ontario)) | Not applicable | All women had given birth in the previous two years  All women were of relatively low socioeconomic status.  Marital status: married (9); divorced (1); single (1)  Education: grade 5 (2); grade 7 (1); grade 8 (1); grade 9 (1); grade 12 (4); college (1); unknown (1)  Employment: bookkeeper (1); dressmaker (3); nail technician (1); stayed at home (5); unknown (1)  Years in Canada: 2 years (1); 4 years (2); 6 years (1); 7 years (1); 9 years (2); 10 years (1), 12.5 years (1); 14 years (1); unknown (1) | Vietnamese women;  1) Language (poor comprehension of English) 2) Transport (no access to automobiles) 3) Time constraints (too busy at home or at work) 4) Never heard about prenatal support services (e.g. prenatal care) | Vietnamese women:  1) Prenatal classes conducted in Vietnamese 2) Prenatal support by trained Vietnamese health workers or peers 3) Audio-visual material instead of written material |
| # 28: McAree et al. 2010 (United Kingdom) | Not applicable | The women interviewed gave birth two to three years ago | Not reported | Indian, Sri Lankan, Pakistani women:  Group prenatal care classes are more accessible when practice midwives speak several community languages. |
| # 29: Essén et al. 2000 (Sweden) | Not applicable | Time elapsed since last birth was between 6 months and 7 years.  Two women had given birth only in Somalia, five only in Sweden, and eight in both Somalia and Sweden  The women migrated to Sweden between 1989 and 1995 | Somali women:  Not understanding why the antenatal care practice should be viewed as necessary, as pregnancy is seen as a normal healthy state | Not reported |
| # 30: Bhagat et al. 2002 (Canada (Lower mainland of British Columbia)) | Not applicable | Not reported | Punjabi (Indian) women:  1) Lack of knowledge about the Western health care system 2) Lack of knowledge about the importance of prenatal care 3) Language barrier: the need to have some English to use while in hospital 4) No time to attend six prenatal classes | Punjabi (Indian) women:  1) The health care provider facilitating the groups should be a Punjabi speaking women with childbirth experience 2) For women to attend prenatal classes, prenatal care needs to be recognized as an important issue in the community. The community should be mobilized through three channels: word of mouth, radio and television 3) Renaming prenatal classes to prenatal sessions |
| # 31: Bollini et al. 2007 (Switzerland (La-Chaux-de-Fonds; Bern; Zurich; Fribourg)) | Not applicable | Have at least one pregnancy in Switzerland  Not married or living with a Swiss national  Marital status: Single (1); Married (10); Separated/divorced (3)  Current occupation: Housewife (7); Unskilled manual work (5); Clerical work (1); Professionals (1)  Education: Primary school or less (6); Secondary school (3); Professional training (2); University or equivalent diploma (3)  Length of stay in Switzerland: <5 years (2); 5-14 years (6); >14 years (6) | Turkish women:  1) Not attending prenatal courses because of language problems and lack of proper information 2) Not aware of the need to regularly consult a gynaecologist during pregnancy 3) Problems obtaining appropriate medical leave from work 4) Poor socioeconomic conditions which affected the ability to pay for health insurance, especially during the initial years in Switzerland | Turkish women:  1) More information in women’s own language 2) More efforts by the institutes to improve communication 3) Better socioeconomic follow up |
| # 32: Davies and Bath 2001 (the United Kingdom (in a northern English city)) | Not applicable | Living in a Northern English city  Having used maternity and women's health services in this Northern English city  One woman did not have children, while another woman had given birth in the Netherlands shortly before arriving in the UK | Somali women:  1) Unfamiliarity with the multi-layered organization structure of the National Health Service (NHS). The general practitioner is the only point of reference 2) Language barrier and poor communication with healthcare professionals 3) Booking appointments three days in advance if the interpreting service is needed. Because of this, contact with health professionals is often delayed 4) Sometimes refused to be seen by a general practitioner if someone who could provide interpretation is not brought along | Not reported |
| # 33: Binder et al. United 2012 Kingdom (Greater London) | Not applicable | Time spent in the United Kingdom ranged from 1 to 20 years for the Somali and Ghanaian women  All women interviewed had at least one child within the British health care system  All women interviewed were living in Greater London at the time of data collection | Somali women:  Perceiving yourself as having been badly treated by a care provider | Not mentioned |
| # 34: Rice and Naksook 1998 (Australia (Melbourne)) | Not applicable | Religion: Buddhist (30)  Education level: primary (11); secondary (5); tertiary (13); no education (1)  Occupation: home duties (15); self-employed (5); office based (4); casual (2); part-time (3); pension (1)  Marital status: Married (25); divorced (1), separated (1); widowed (1); single mother (2)    Length of stay in Australia: 1-5 years (16); 6-10 years (12); 11-15 years (1); 16+ years (1)  Husband's ethnic background: Anglo-Australian (8); European English speaking (6); other European (4); Thai (6); Other Asian (5); Middle East (1)  Length of marriage: 0-5 years (19); 6-10 years (6); 11-15 years (3); 16+ years (2) | Thai women:  1) Already experienced birth in Thailand 2) Husband was working and did not know how to get to the hospital by herself 3) Feelings of fear and embarrassment to watch a video to prepare for birth | Thai women:  1) Antenatal care is seen as an important aspect of pregnancy which can assure women about their baby's well-being 2) Believing in looking after your own health and body, and wanting everything about the baby to be as perfect as possible 3) Being worried about the baby's health 4) Husband with an English speaking background who told them to go for antenatal check-ups and birth |
| # 35: Baken et al. 2007 (Italy (Cesena area))  Only the qualitative part is included in this review! | Not applicable | Not reported | Chinese women, Northwest African women (Maghreb):  1) Communication problems due to language 2) Lack of information about services in Italy 3) Logistic problems: transport, opening hours (incompatible with women’s own working hours, their husbands’ or accompanying persons’) 4) Little or no support from family 5) Social inequalities (education, economic resources and residence (rural or urban)) 6) Lack of autonomy and dependency of women on their husband (Northwest African women) 7) Isolation of the community (Chinese women) | Not mentioned |
